# Ground Reaction Forces and Joint Moments Predict Metabolic Cost in Physical Performance: Harnessing the Power of Artificial Neural Networks

**DOI:** 10.3390/app14125210

**Published:** 2024-06-15

**Authors:** Arash Mohammadzadeh Gonabadi, Farahnaz Fallahtafti, Prokopios Antonellis, Iraklis I. Pipinos, Sara A. Myers

**Affiliations:** 1 Department of Biomechanics and Center for Research in Human Movement Variability, University of Nebraska at Omaha, Omaha, NE 68182, USA;; 2 Institute for Rehabilitation Science and Engineering, Madonna Rehabilitation Hospitals, Lincoln, NE 68506, USA; 3 Department of Neurology, Oregon Health & Science University, Portland, OR 97239, USA; 4 Department of Surgery, University of Nebraska Medical Center, Omaha, NE 68105, USA;; 5 Department of Surgery and Research Service, Nebraska-Western Iowa Veterans Affairs Medical Center, Omaha, NE 68105, USA

**Keywords:** metabolic cost, artificial neural networks, biomechanics, gait, ground reaction forces, joint moments, human movement analysis

## Abstract

Understanding metabolic cost through biomechanical data, including ground reaction forces (GRFs) and joint moments, is vital for health, sports, and rehabilitation. The long stabilization time (2–5 min) of indirect calorimetry poses challenges in prolonged tests. This study investigated using artificial neural networks (ANNs) to predict metabolic costs from the GRF and joint moment time series. Data from 20 participants collected over 270 walking trials, including the GRF and joint moments, formed a detailed dataset. Two ANN models were crafted, net_GRF_ for the GRF and net_Moment_ for joint moments, and both underwent training, validation, and testing to validate their predictive accuracy for metabolic cost. Net_GRF_ (six hidden layers, two input delays) showed significant correlations: 0.963 (training), 0.927 (validation), 0.883 (testing), *p* < 0.001. Net_Moment_ (three hidden layers, one input delay) had correlations of 0.920 (training), 0.956 (validation), 0.874 (testing), *p* < 0.001. The models’ low mean squared errors reflect their precision. Using Partial Dependence Plots, we demonstrated how gait cycle phases affect metabolic cost predictions, pinpointing key phases. Our findings show that the GRF and joint moments data can accurately predict metabolic costs via ANN models, with net_GRF_ being notably consistent. This emphasizes ANNs’ role in biomechanics as a crucial method for estimating metabolic costs, impacting sports science, rehabilitation, assistive technology development, and fostering personalized advancements.

## 1. Introduction

Measuring the metabolic cost from biomechanical data, like ground reaction forces and joint moments, is vital for enhancing health, rehabilitation, and sports through human movement science [1]. Traditional methods need up to five minutes to average metabolic cost data, but recent advances have cut this to about two minutes [2]. However, these techniques still cannot quickly assess metabolic costs during a gait cycle, posing challenges for patients in lengthy walking tests.

The significance of ground reaction forces (GRFs) in biomechanical analysis is well-documented, with methodologies ranging from direct force plate measurements to sophisticated estimations using wearable sensors and inertial motion capture systems [3]. Such advancements have democratized gait and movement analysis, extending the reach of biomechanical assessments beyond the confines of specialized laboratories [4]. Similarly, joint moments offer a window into the internal dynamics of the musculoskeletal system, revealing the intricate interplay between muscle forces and the resulting movements [4,5]. Recent research has further pushed the boundaries of biomechanical analysis by integrating these metrics with advanced computational models to predict metabolic cost [5–9]. This interdisciplinary approach melds the precision of biomechanical measurements with the predictive power of computational algorithms, unveiling new perspectives on the energetics of human movement. The use of neural networks, in particular, exemplifies this trend, showcasing the potential of machine learning in deciphering complex relationships between biomechanical inputs and metabolic outcomes [10].

Drawing from a broader spectrum of artificial intelligence (AI) applications, our previous work has demonstrated the efficacy of optimization methods and artificial neural networks (ANNs) across diverse domains [11–13]. These studies underscore AI techniques’ adaptability and bring novel insights into biomechanical analysis. A unified model was developed that leverages walking mechanics to estimate metabolic costs under various conditions, demonstrating the potential of using biomechanical measurements, such as the GRF and joint moments, to predict metabolic costs in human walking [1]. The model’s capability to accommodate different walking conditions underscores its versatility and applicability in biomechanical analysis, offering new insights into the energetic efficiency of gait. Another study [14] compared metabolic cost calculations in gait using musculoskeletal energy models. They evaluated the correlation of several metabolic energy expenditure models with experimental data, enriching our understanding of how different models predict the metabolic costs of gait under varied conditions. Integrating the GRF, marker data, and pulmonary gas exchange measurements in their methodology provides a comprehensive approach to assessing the metabolic implications of gait dynamics.

By synthesizing insights from multiple studies, our research underscores the value of merging biomechanical data with AI models to predict metabolic costs. It leverages AI to evaluate how the GRF and joint moments data forecast metabolic costs, enhancing our theoretical and practical understanding of biomechanical energetics. This work paves the way for developing interventions and devices to improve movement efficiency and health.

ANNs are computational models inspired by the human brain’s structure and function [15,16]. Recent research has leveraged ANNs to gain deeper insights into the mechanics of movement and the prediction of metabolic costs, offering substantial advancements in personalized healthcare and athletic performance optimization. For instance, a literature review [17] demonstrated the application of ANNs in predicting the metabolic cost associated with different physical activities, showcasing the potential of machine learning in enhancing our understanding of energy expenditure in human motion.

Machine learning algorithms are widely used to analyze joint kinetics, providing a novel approach to assessing the biomechanical factors influencing athletic performance and injury risk [18]. Furthermore, an editorial section in ‘Frontiers in Sports and Active Living’ [19] discussed the integration of AI and machine learning in biomechanical research, highlighting how these technologies are reshaping the analysis and interpretation of complex biomechanical data. The incorporation of ANN models enables a more nuanced understanding of human movement, facilitating the development of targeted interventions and assistive technologies designed to optimize movement efficiency and reduce the risk of injury. Expanding on Takallou et al.’s work [20], this research explores the potential of deep learning to elucidate complex biomechanical data connections. Their study, centered on distinguishing individuals with peripheral artery disease through gait analysis using acceleration data, exemplifies AI’s broad applicability, including biomechanics to support clinical decision making.

Based on the established groundwork, our research introduces two main hypotheses to advance the investigation into estimating metabolic costs using biomechanical data. First, we hypothesize that GRF signals, when analyzed through ANNs, can accurately estimate the metabolic cost associated with human locomotion. This hypothesis is predicated on the integral role that the GRF plays in reflecting the external forces acting on the body during movement, which are closely linked to the energetic demands of such activities. Second, we propose that joint moments—quantitative measures of the forces acting around the joints—can also be reliable predictors of metabolic cost when processed through an ANN. This hypothesis stems from the understanding that joint moments enclose the internal biomechanical dynamics, including muscle forces and their lever arms, which are key determinants of the metabolic energy required for movement. Our study rigorously tested these hypotheses to compare the effectiveness of the GRF and joint moments data in predicting metabolic cost, thus enriching our understanding of energy efficiency in human movement.

## 2. Materials and Methods

### 2.1. Data Sources and Preprocessing

This study incorporated the time series data from two distinct research projects, each with 10 different participants, exploring various aspects of human gait over treadmill walking. The ‘Footwear Study’ focused on the effects of shoe design on gait under 15 conditions, using 200 Hz for motion capture and 2000 Hz for GRF data [21]. In this study, ten healthy male participants (age 24.9 ± 2.7 years, mass 75.7 ± 13.4 kg, height 173.2 ± 6.4 cm; mean ± s.d.) were recruited. The ‘Hip Exoskeleton Study’ assessed the biomechanical impact of hip exoskeletons under 12 conditions, using 120 Hz for motion capture and 1000 Hz for GRF data acquisition [22]. In this study, ten healthy adults (four males, six females; age: 27.6 ± 5.9 years, body mass: 65.3 ± 13.1 kg, height: 1.66 ± 0.08 m) participated. Both experiments were performed at a constant walking speed of 1.25 m/s on a treadmill. One dataset included variations in outsole geometry, slopes, and treadmill inclination, while the other involved walking with an exoskeleton providing different hip assistance timings. The equipment used included a bilateral semi-rigid exoskeleton for hip motion assistance, and precise sensor calibration ensured high accuracy in data collection. In both studies, the net metabolic rate for walking, expressed in watts per kilogram (W/kg), was determined by the indirect calorimetry method [23]. These computed metabolic cost values serve as the ‘target values’ in our research. Combining the two studies, 270 trials of biomechanical data were collected, including the GRF, normalized as N/kg, and joint moments, normalized as N·m/kg for biological relevance. Data were preprocessed to calculate stride-average time series, ensuring consistency and comparability across the various trials and conditions. To maintain uniformity and ensure comparability across the 270 trials, we standardized the sample rates; GRF signals were normalized to a sample rate of 1000 Hz and motion capture data to 120 Hz. Additionally, we transformed each time series from the trials into 100 data points, reflecting the percentage of the gait cycle. This approach ensured consistency in data analysis and allowed us to focus on the biological moments in the joint moment time series, providing a clear and comparable biomechanical insight across the 270 trials from the two distinct studies.

### 2.2. Neural Network Design and Implementation

Our analytical framework primarily leverages ANNs, specifically Nonlinear Autoregressive Networks with Exogenous Inputs (NARX) [16], to derive metabolic costs from biomechanical time series data. Our choice to utilize ANNs, particularly NARX, over traditional statistical methods in this investigation is driven by their superior capability in modeling complex nonlinear dynamics often inherent in time series datasets, such as those encountered in biomechanical data analysis [15]. In ANNs, ‘hidden layers’ are intermediary layers between the input and output, crucial for learning complex data patterns. These layers do not interact directly with the input or output but process and transform the information, allowing the ANN to handle intricate problems. ‘Delays’ in the context of ANNs, mainly when dealing with time series data, are essentially a way to incorporate historical information into the model’s current prediction. The concept revolves around using data from previous time steps as input for predicting future outcomes.

In our investigation, we operationalized two distinct NARX models tailored to the two types of biomechanical data: the GRF and joint moments. This bifurcation was strategically aimed at harnessing the specific strengths of NARX networks in handling the diverse complexities inherent in each data type [16]. Each NARX network was configured with an input layer tailored to accommodate the stride-averaged time series data, hidden layers whose size was optimized based on preliminary trials to balance the trade-off between model complexity and predictive accuracy, and an output layer designed to estimate the metabolic cost. The networks underwent rigorous training, validation, and testing processes, utilizing a subset of the compiled dataset, with performance metrics, such as the mean squared error (MSE) and correlation coefficients (R-values), serving as key evaluative criteria [24].

**Figure 1.**
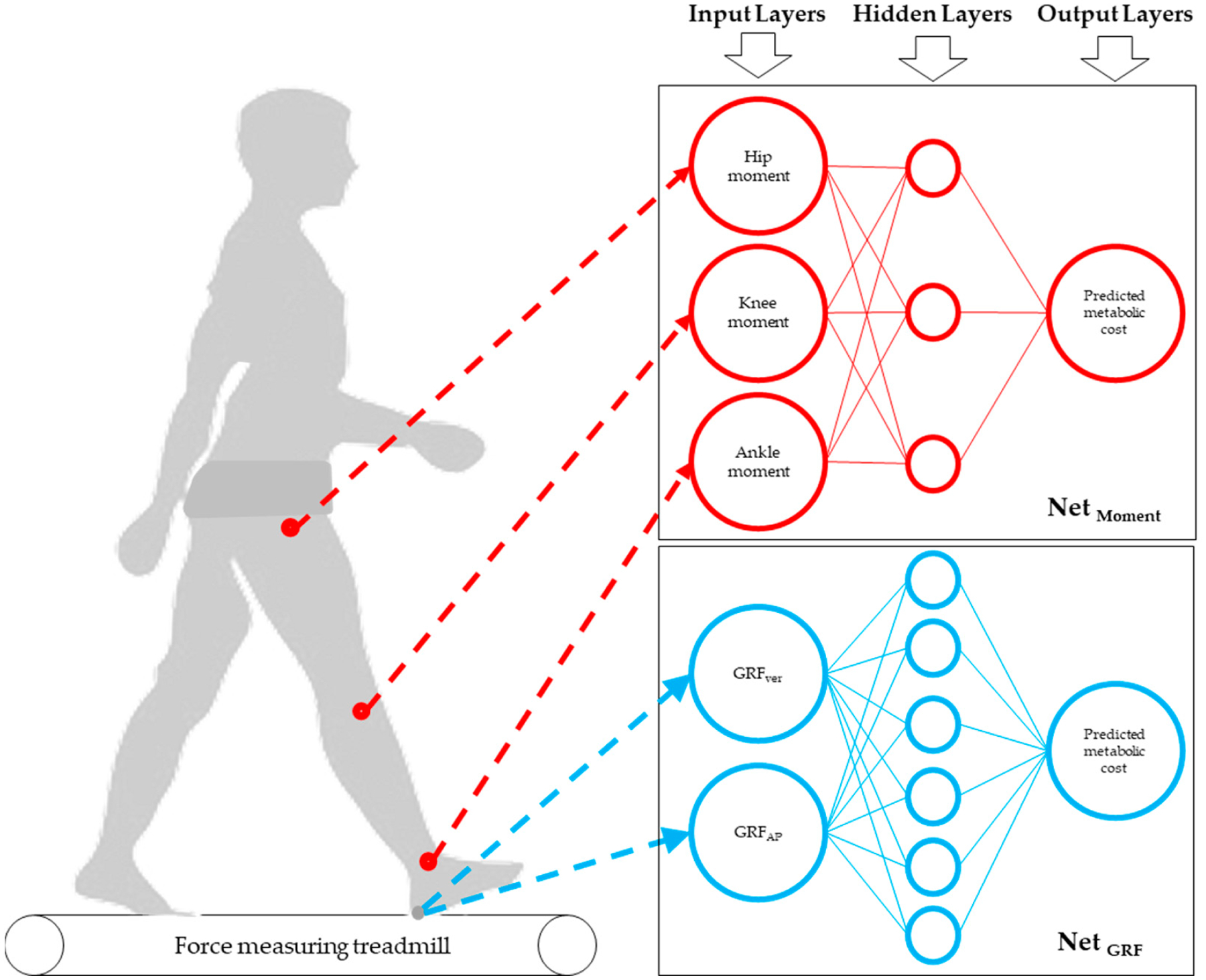
The architecture of the neural network model. This schematic represents the ANN models for predicting metabolic cost from biomechanical data. The upper network illustrates the model using hip, knee, and ankle moments as inputs to predict metabolic cost. The lower network demonstrates the model utilizing vertical (GRF_ver_) and anterior–posterior (GRF_AP_) ground reaction forces as inputs to predict metabolic cost. Both models highlight the data flow from the input through the hidden layers to the output, depicting the ANN’s ability to process complex biomechanical inputs to predict energetic expenditure.

We developed two distinct ANN models using time series data from the GRF and joint moments across a dataset of 270 trials (Figure 1). We selected a 70% (188 trials) training, 15% validation (41 trials), and 15% testing (41 unseen trials) split for each model to ensure a robust learning foundation while maintaining enough data for meaningful validation and testing. The NARX model was trained using the Levenberg–Marquardt backpropagation algorithm. The training process utilized a learning rate of 0.001, a batch size of 15, and 1000 epochs.

### 2.3. Neural Network Optimization Process

To enhance the robustness of our NARX neural network models, we adopted an exhaustive optimization strategy inspired by Nogueira et al. [25]. This involved nested for-loops to systematically explore various network configurations, focusing on hidden layer size (ranging from 2 to 50 to evaluate network complexity’s impact on accuracy), delays (adjusted from 1 to 4 to capture temporal dependencies), and model reiterations (repeating the ANN 1000 times for each parameter combination to ensure stability and minimize stochastic variations). Each configuration was rigorously evaluated based on predefined performance metrics, including the MSE, for training, validation, test sets, and R-values to assess the model’s predictive accuracy. This systematic exploration ensured that the final selected models were optimal regarding network architecture and demonstrated reliable performance across multiple iterations.

### 2.4. Computational Implementation

The entire optimization process was implemented in MATLAB (MathWorks, Natick, MA, USA), utilizing its neural network toolbox for model construction and training, along with a customized MATLAB code we developed for this study.

### 2.5. Partial Dependence Plots (PDPs)

PDPs are a valuable tool in model interpretation, providing insights into the relationship between the predictors and the response variable in machine learning [26–28]. We identified which phases are most influential by adjusting the value of one gait cycle percentage at a time and observing the Root-Mean-Square Error (RMSE) changes. The plots generated reveal the dependency of the predicted metabolic cost on the selected gait cycle percentage, providing a clear visual representation of how changes in the gait cycle correlate with prediction accuracy. This method is applied to various models based on the GRF and joint moments to uncover which gait cycle phases are critical for accurate metabolic cost estimation.

### 2.6. Statistical Analyses

The statistical analysis focused on evaluating the accuracy and reliability of the ANN models by comparing the predicted metabolic cost to the actual data. This involved assessing the model’s performance in terms of error distribution, response correlation, and predictive strength across different phases of data (training, validation, and testing). The analysis also included examining patterns in the prediction errors (MSE) and their correlation with input variables, providing insights into the model’s behavior and areas for improvement. To provide a comprehensive evaluation of our ANN models, we expanded our assessment metrics to include accuracy [29], recall, precision, F1 score [30], and k-fold cross-validation (with k = 5) [31]. Regression analysis further quantified the degree to which the model’s outputs aligned with the target values, offering a comprehensive overview of its effectiveness. We performed all statistical analyses in MATLAB. For all the statistical tests, we set the significance threshold at 0.05.

## 3. Results

**Figure 2.**
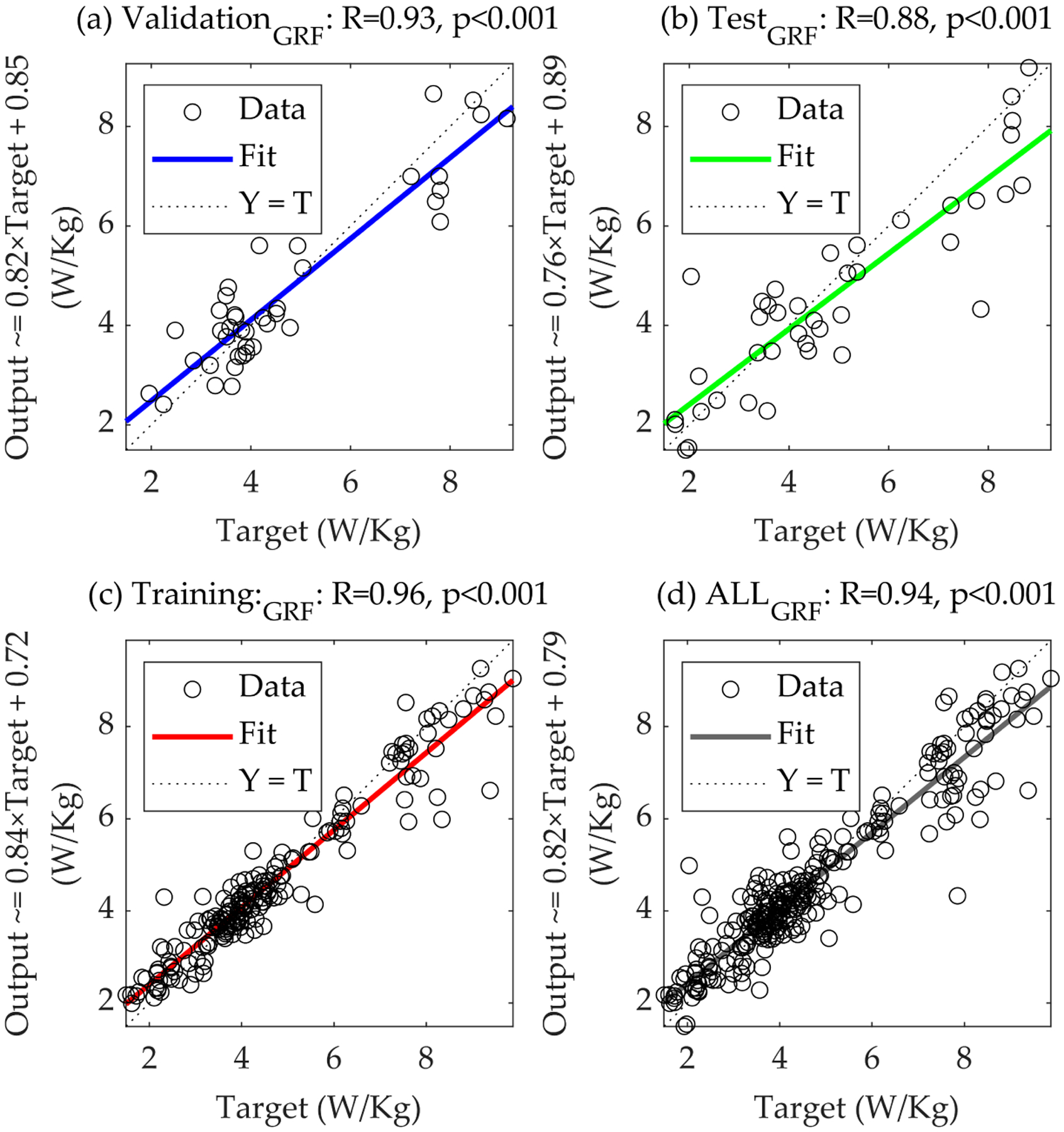
Comprehensive regression analysis of the net_GRF_ model. This figure delineates the regression plots for the net_GRF_ model across (**a**) ‘validation’, (**b**) ‘test’, (**c**) ‘training’, and (**d**) ‘all’ datasets, illustrating the model’s predictive accuracy and generalization capability. The plots compare the predicted metabolic costs against actual values, with each subset providing insights into the model’s performance in different phases of data handling, from learning to generalization on unseen data.

The net_GRF_, with a hidden layer size of six and input delays set to two, achieved significant (*p*-value < 0.001) strong R-values across training, validation, and testing datasets, with the R-value of the training at 0.962, the R-value of the validation at 0.937, and the R-value of the test at 0.883 (Figure 2 and Table 1). These values indicate a robust correlation between the predicted and actual metabolic costs, underscoring the model’s efficacy. The MSEs were notably low, with training performance at 0.0036, validation performance at 0.0064, and test performance at 0.0140 in (W/kg)^2^, further affirming the model’s precision (Table 1).

**Figure 3.**
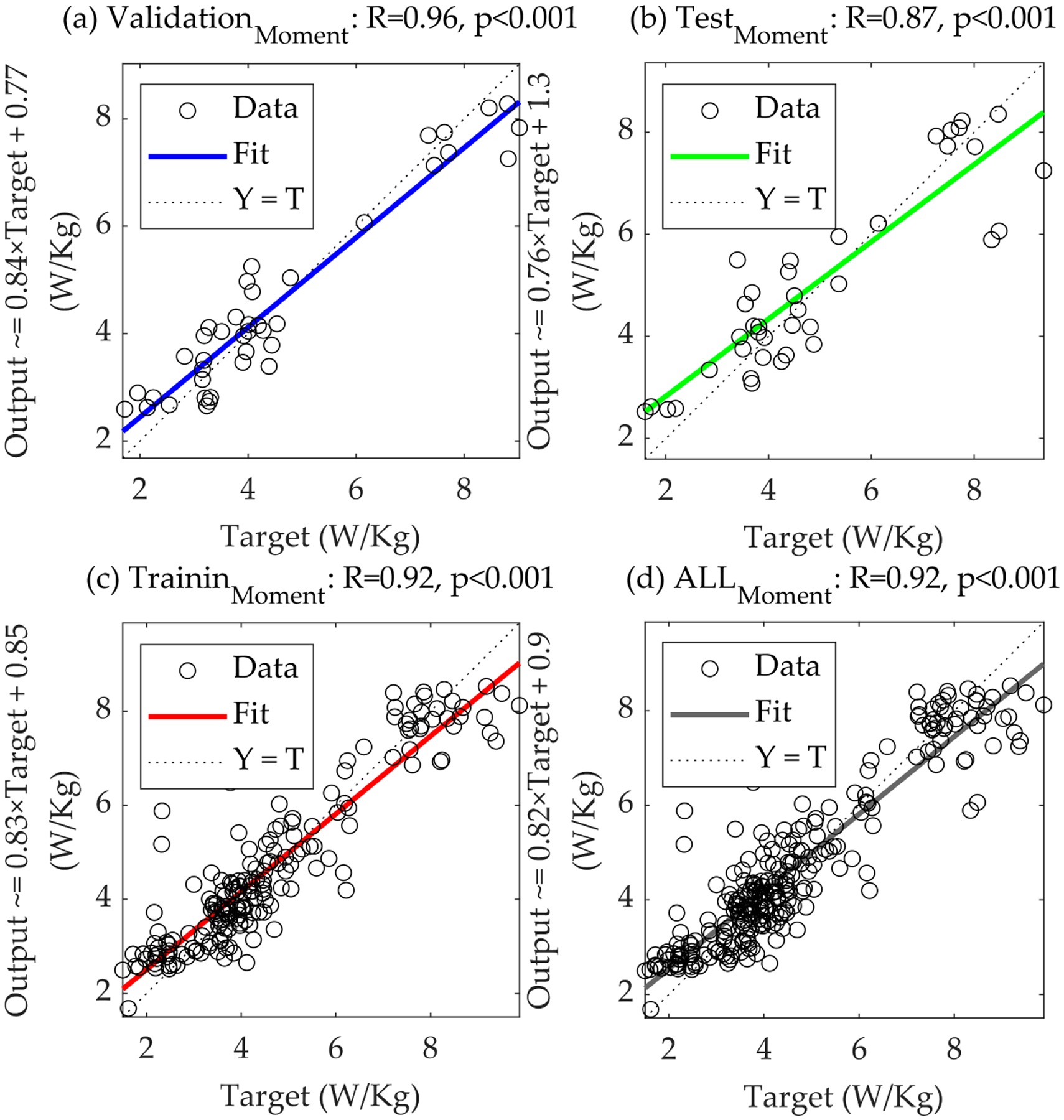
Comprehensive regression analysis of the net_Moment_ model. This figure delineates the regression plots for the net_Moment_ model across (**a**) ‘validation’, (**b**) ‘test’, (**c**) ‘training’, and (**d**) ‘all’ datasets, illustrating the model’s predictive accuracy and generalization capability. The plots compare the predicted metabolic costs against actual values, with each subset providing insights into the model’s performance in different phases of data handling, from learning to generalization on unseen data.

The joint moments ANN model (net_Moment_), with a more streamlined architecture featuring a hidden layer size of three and input delays of one, likewise exhibited solid predictive capabilities. The R-values for this model were similarly solid and significant (*p*-value < 0.001), with the R-value of the training reaching 0.920, the R-value of the validation at 0.956, and the R-value of the test at 0.874 (Figure 3 and Table 1). These results highlight the model’s ability to estimate metabolic cost from joint moments data accurately.

**Figure 4.**
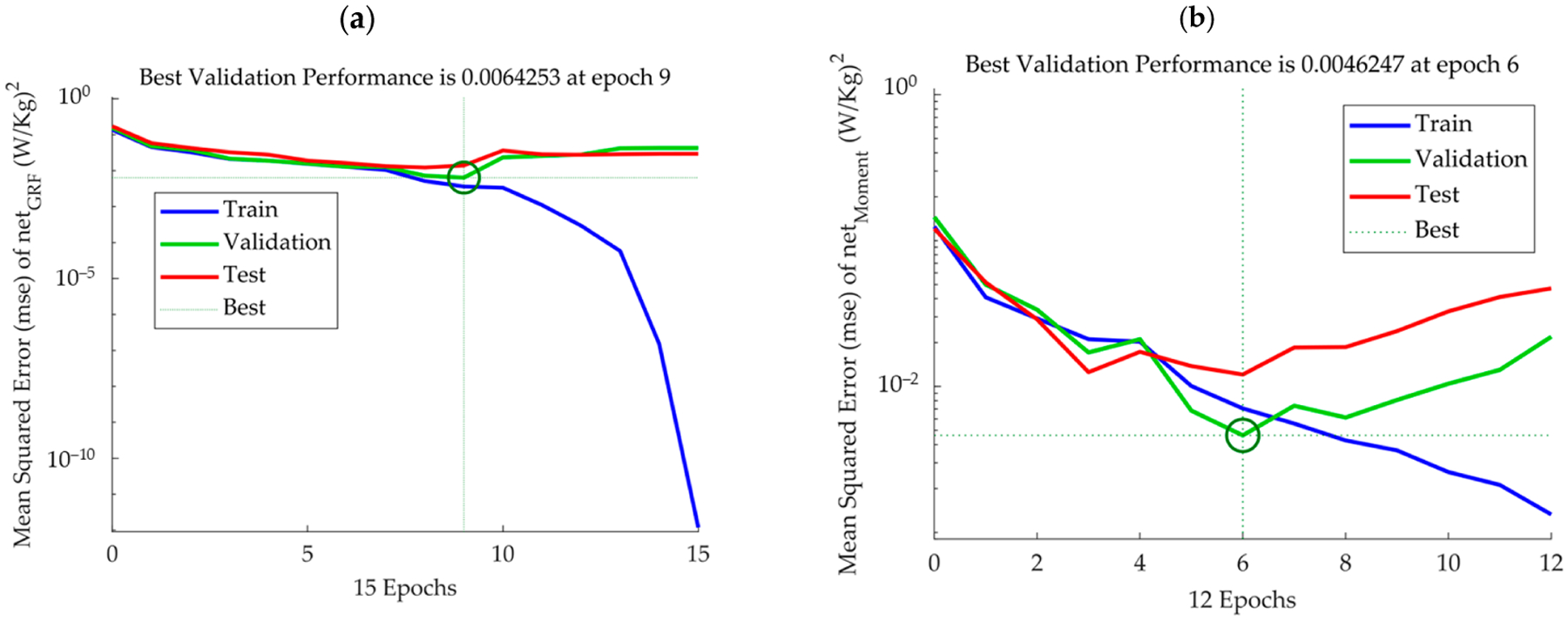
Performance of the ANN models for the GRF and joint moments. Panel (**a**) illustrates the training and validation performance over epochs for the net_GRF_ model, while Panel (**b**) does the same for the net_Moment_ model. These plots showcase the models’ learning progress, highlighting moments of optimal learning and potential overfitting through changes in error rates and performance metrics. The green circle highlights optimal learning epochs and signs of overfitting.

The net_Momen_ model showed strong performance, achieving training, validation, and test scores of 0.0071, 0.0046, and 0.0121 in (W/kg)^2^, respectively, underscoring its accuracy (Figure 4b and Table 1). An ‘epoch’ in machine learning, specifically in training ANNs, refers to one complete pass of the entire training dataset through the network [15,16]. The green circle in Figure 4a,b symbolizes the epoch at which the model’s validation performance is at its peak.

**Figure 5.**
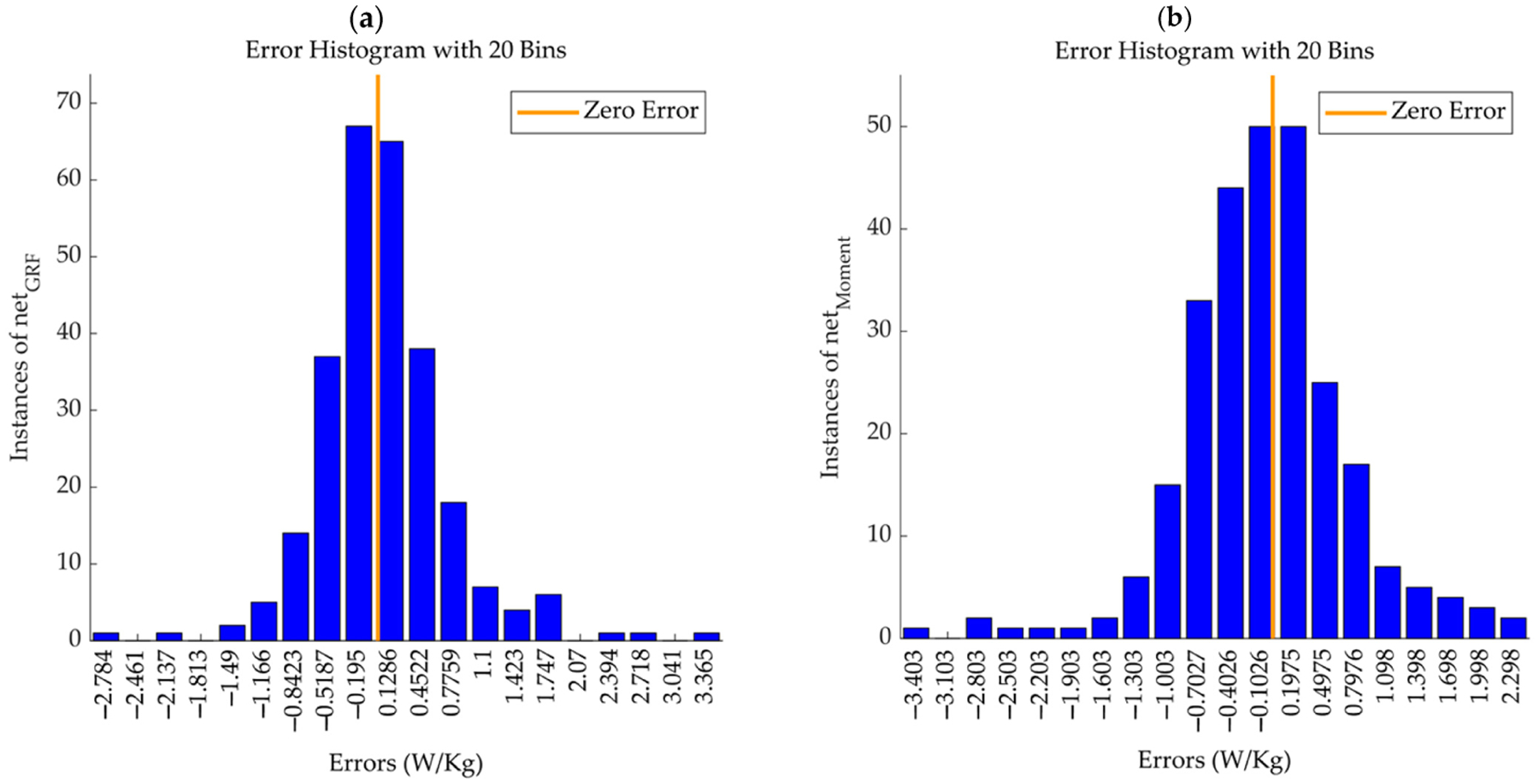
Error histograms for the GRF and joint moment models. Panel (**a**) presents the error distribution for net_GRF_ model, and Panel (**b**), for the net_Moment_ model, illustrates the variance between predicted and actual metabolic costs. The histograms elucidate the frequency of error magnitudes, aiding in identifying consistent prediction deviations and outlier errors for model refinement. A concentration of errors closer to zero for the net_GRF_ model indicates a tighter clustering of predicted values around the actual metabolic costs and, consequently, a higher accuracy. In contrast, there is a broader spread of errors for the net_Moment_ model, implying a greater variance in the accuracy of predictions.

Figure 5 demonstrates the GRF and joint moment model error histograms (Figures 5a and 5b, respectively). Comparing the two models, both demonstrated exceptional predictive abilities, as evidenced by their high R-values and low MSEs. The GRF model, despite its more complex network structure, did not significantly outperform the joint moments model in terms of R-values, suggesting that both the GRF and joint moments are valuable predictors of metabolic cost. However, the GRF model showed slightly better consistency across different performance metrics (MSEs and R-values), potentially offering a more versatile tool for estimating metabolic cost in varied applications.

**Figure 6.**
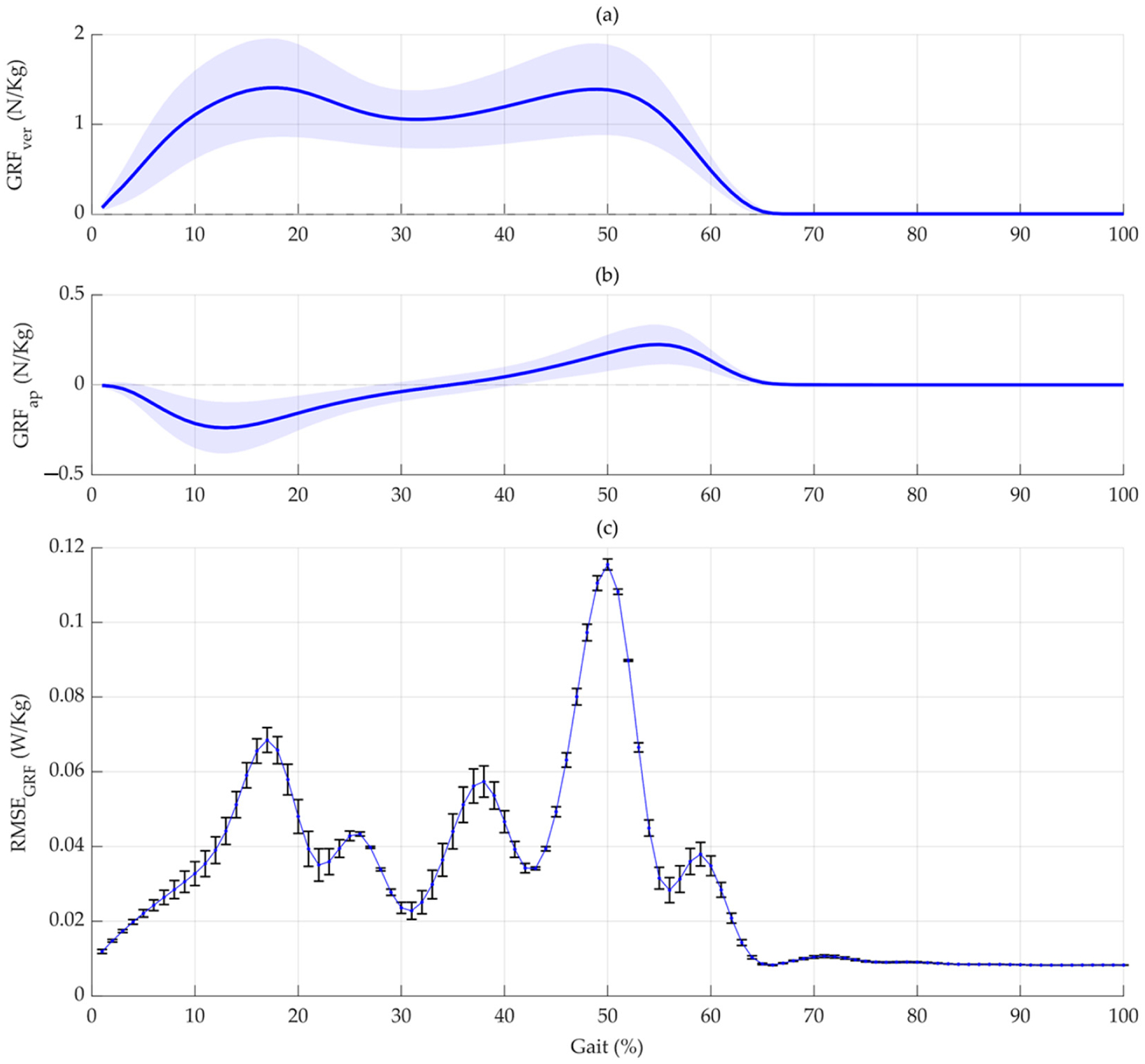
Analysis of ground reaction force influences on metabolic cost prediction under net_GRF_. (**a**) Displays the vertical ground reaction force (GRF_Ver_) signals throughout various percentages of the gait cycle, aggregated across all trials (270 in total). (**b**) Shows the progression of anterior–posterior ground reaction force (GRF_AP_) signals over the gait cycle and across all trials. (**c**) Presents a detailed view of how each percentage of the gait cycle impacts the Root-Mean-Square Error (RMSE) between the predicted and actual metabolic costs under net_GRF_. This visualization, the Partial Dependence Plot (PDP), underscores the significance of distinct phases of the gait cycle in the model’s prediction accuracy. Vertical lines represent the standard deviation of the RMSE, indicating variability and confidence at each point in the gait cycle. The constancy of other factors during this analysis suggests these influences are directly attributable to variations in the gait cycle’s ground reaction forces.

**Figure 7.**
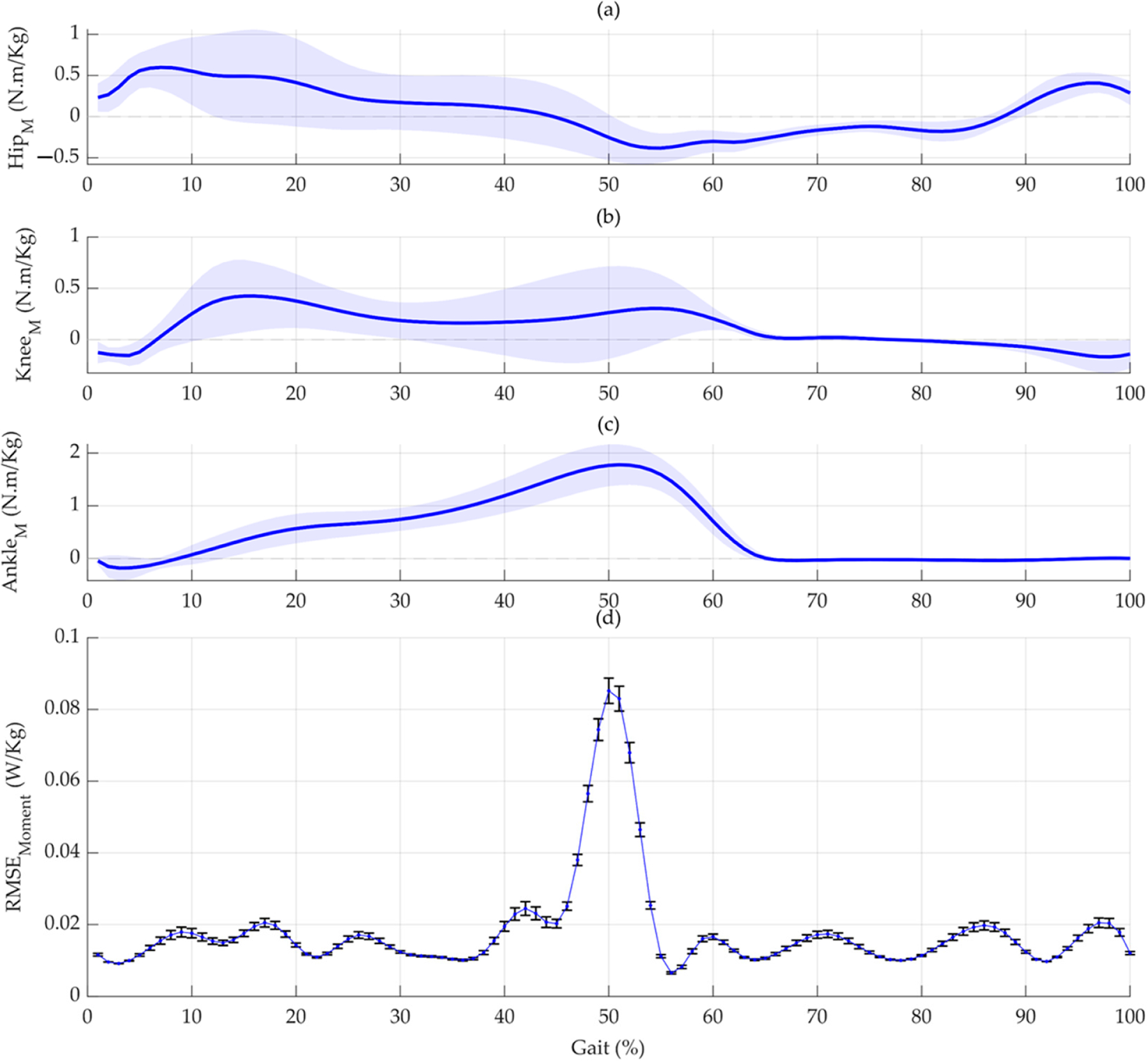
Analysis of joint moments’ influences on metabolic cost prediction under net_moment_. (**a**) Depicts the hip joint moment (Hip_M_) variations across different gait cycle percentages, compiled from all trials (270 in total). (**b**) Illustrates the progression of knee joint moment (Knee_M_) signals throughout the gait cycle, encompassing all trials. (**c**) Portrays ankle joint moment (Ankle_M_) fluctuations over the gait cycle and aggregates data from all trials. (**d**) Provides a granular analysis of each gait cycle percentage’s influence on the Root-Mean-Square Error (RMSE) between the model’s predicted and the actual joint moments under net_moment_. This plot, the Partial Dependence Plot (PDP), highlights the critical phases of the gait cycle that significantly affect the model’s accuracy in predicting joint moments. The vertical lines indicate the standard deviation of the RMSE, offering insights into the variability and reliability of the model’s predictions at each gait cycle percentage. The stability of other variables in this analysis implies that these effects are directly linked to changes in the joint moments during the gait cycle.

To further our understanding of how specific phases within the gait cycle influence metabolic cost predictions, we employed the PDPs [26–28]. This analysis was applied to GRF-based and moment-based networks to discern which gait cycle phases exert a more pronounced effect on the models’ predictive accuracy, as quantified by the RMSE. For the GRF-based network, we systematically varied the GRF vertical and anterior–posterior signals across different gait cycle percentages from zero to their maximum observed values. By isolating one percentage at a time and observing the resultant changes in the RMSE, we could pinpoint phases of the gait cycle that are critical for accurate metabolic cost estimation. The first two plots focus on the GRF vertical and AP directions, respectively (Figure 6). Similarly, an analogous approach was undertaken for the moment-based network (Figure 7).

## 4. Discussion

Our study aimed to advance the understanding of metabolic cost estimation through biomechanical data, focusing on the predictive capabilities of the GRF and joint moments using ANNs. We hypothesized that both the GRF and joint moments could accurately estimate metabolic costs. Our objective was to assess their comparative predictive power, offering insights into the biomechanics of energy efficiency in human locomotion. Our results supported these hypotheses, as the results indicate that both the GRF and moment ANN models can predict metabolic cost. The comparative analysis reveals that the net_GRF_ model, with more neurons in the hidden layer, showed slightly better overall performance and higher R-value of the training component (*p*-value < 0.001) than the net_Moment_ model. However, the net_Moment_ model demonstrated a more robust correlation and significant R-value for the validation component (*p*-value < 0.001), indicating potential differences in generalization capabilities. The net_GRF_ model resulted in a higher test performance error, suggesting possible overfitting compared to the net_Moment_ model, which had a more balanced error distribution across training, validation, and testing phases (Table 1).

Determining the ‘best’ model between net_GRF_ and net_Moment_ depends on specific criteria and application needs. If predictive accuracy and lower training errors are prioritized, the net_GRF_ model might be preferred due to its significant, stronger (*p*-value < 0.001) R-value in training and overall performance. However, for applications valuing generalization to unseen data, the net_Moment_ model, with its stronger, significant validation (*p*-value < 0.001) R-value, could be more suitable, despite its slightly lower overall R-value. The choice ultimately hinges on whether the focus is on training accuracy or validation performance.

The GRF model demonstrates higher accuracy and superior performance across the other evaluation metrics (accuracy, recall, precision, F1 score, and k-fold cross-validation) compared to the moment prediction model (Table 1). This indicates that the GRF model provides more reliable and precise predictions, making it a more effective tool for our study’s objectives.

The regression analysis for the net_GRF_ model across ‘validation’, ‘test’, ‘training’, and ‘all’ datasets (Figure 2) illustrates a robust linear relationship between the predicted and actual metabolic costs, with the R-values serving as a quantitative measure of this relationship. Strong R-values across all subsets suggest that the net_GRF_ model has learned the underlying patterns effectively and can generalize well to new data. The closeness of data points to the line where predicted values match actual values in the plots signifies the model’s precision. This is particularly evident in the validation subset, where a close alignment highlights the model’s accuracy with validation data. Similarly, the regression analysis for the net_Moment_ model (Figure 3) demonstrates that while the overall trend of the data points suggests a reasonable fit, the spread of the points around the line of unity is more pronounced than in the net_GRF_ model, especially in the ‘test’ subset, as indicated by the lower R-value. This suggests that while the net_Moment_ model has learned to an extent, its predictive performance may not be as robust as the net_GRF_ model when applied to new data.

Figures 2 and 3 underscore the nuanced differences in the performance of the two models. The net_GRF_ model’s stronger R-values imply a more reliable predictive model for estimating metabolic costs from biomechanical data. The data points’ distribution to the fit line and the line of unity across all plots serves as a graphical testament to the net_GRF_ model’s superior ability to capture the essential patterns of the underlying biomechanical processes.

In Figure 4, the training and validation performance plots for the net_GRF_ and net_Moment_ models offer critical insights into the learning dynamics of each network. Figure 4a demonstrates a consistent decrease in the MSE for the net_GRF_ model as the number of epochs increases, with the best validation performance occurring at epoch 9. This suggests that the net_GRF_ model is effectively learning the underlying patterns in the data without signs of significant overfitting, as indicated by the convergence of training and validation error rates. Figure 4b, on the other hand, illustrates the net_Moment_ model’s learning progression, with the best validation performance achieved at an earlier epoch, epoch 6. The green circle indicates a key moment where the validation error minimizes before the test error rises, which may signal the onset of overfitting. This point is crucial for model tuning, as it identifies the epoch after which the model may start to learn noise from the training data rather than underlying trends, reducing its generalization performance on new data. The comparative analysis of these learning curves informs the model selection process, guiding the implementation of early stopping or other regularization techniques to optimize model performance.

The error histograms presented in Figure 5 for both the net_GRF_ and net_Moment_ models visually represent the variance between predicted and actual metabolic costs. Figure 5a shows a concentration of errors closer to zero for the net_GRF_ model, indicating a tighter clustering of predicted values around the actual metabolic costs and, consequently, a higher accuracy. In contrast, Figure 5b shows a broader spread of errors for the net_Moment_ model, implying a greater variance in the accuracy of predictions. The histograms assist in pinpointing areas where predictions consistently deviate from actual values and identifying outlier errors, which can be pivotal for subsequent model refinements. This graphical analysis of error distribution is instrumental in assessing the predictive performance of the models, guiding efforts to optimize them for enhanced accuracy in estimating metabolic costs.

Our findings, demonstrating a solid predictive relationship between the GRF, biomechanical measurements, and metabolic costs in human walking, align with other studies ([1,32]). The superior performance of our net_GRF_ model, demonstrated by stronger R-values and lower MSEs, resonates with the understanding that the GRF, due to its direct relationship with the external forces during locomotion, can provide a more reliable estimate of metabolic cost compared to joint moments. This is further exemplified in our study, where the net_GRF_ model showed a slightly better consistency across various performance metrics, highlighting its robustness and potential as a versatile tool for biomechanical analysis and intervention design to optimize human movement efficiency.

In the current study, the RMSE across the gait cycle for both the GRF and joint moment models reveals the prediction accuracy at various gait cycle phases. This illustrates the variability in predicted metabolic cost based on biomechanical parameters at different stages of gait. The variability and consistency of these predictions are illustrated through the standard deviation of the RMSE, depicted as vertical lines in Figures 6 and 7. Our models analyze data from various gait cycle phases, enhancing prediction accuracy and providing a complete view of energy efficiency in movement. This approach ensures the models’ effectiveness in real-world scenarios, capturing the full complexity of the gait cycle for precise metabolic cost estimations.

A study by Gonabadi et al. [5] presents the stride average metabolic rate and the time profile of the metabolic rate during different walking conditions. They focused on estimating the stride average metabolic rate and creating a time profile of the metabolic rate during different walking conditions using two methods: musculoskeletal estimation and joint-space estimation. Our study and the previous research found that the push-off phase in the gait cycle generates the largest peaks for both the metabolic cost time profile and the RMSEs. Additionally, a notable peak occurs at approximately 17% of the gait cycle in Figures 6c and 7d, corresponding to the peak in the joint-space model’s metabolic cost time profile in Gonabadi et al.’s study [5]. Moreover, using the musculoskeletal method, the single support phase had the highest metabolic cost. In contrast, the joint-space method estimated the highest metabolic cost during the second double-support phase [5]. Our results are within this range, corresponding to 38% and 50% of the gait cycle (Figures 6c and 7d).

Our method can have practical applications, including identifying and characterizing gait disabilities by pinpointing the most energy-intensive phase of the stride cycle in patients, devising targeted rehabilitation exercises, such as treadmill walking, with robotic resistance during the least metabolically efficient phase of the stride cycle, and contributing to the engineering of efficient assistive devices, like designing an exoskeleton that aids the least economically efficient phase of the stride cycle. It may pose a challenge to characterize the time profile of metabolic cost in individual patients due to limited walking endurance. Therefore, a future challenge involves developing methods that enable the characterization of the time profile of metabolic cost with shorter experimental protocols for patients, potentially by integrating our method with other approaches, such as simulation [5,7,8] or blood flow measurements [33,34]. To address the stability and reliability of our ANN model, we suggest continuous performance monitoring, periodic re-evaluation, and retraining using new data.

This study faces limitations beyond its potential overfitting due to its small sample and potential biases, including the specific design of the ANN models for particular datasets, which may limit their use in broader populations or different biomechanical contexts. The models might also simplify the complex nature of movements, focusing mainly on vertical and parallel GRF signals and lower body joints, potentially neglecting essential elements, like lateral movements and upper body contributions. This could restrict a comprehensive view of biomechanical efficiency and energy use. Furthermore, excluding three-dimensional joint data and other GRF directions rather than vertical and anterior–posterior might be a constraint. This study utilized data from two separate experiments, both conducted at a constant walking speed of 1.25 m/s on a treadmill. One dataset included variations in outsole geometry, slopes, and treadmill inclination, while the other involved walking on a level treadmill with an exoskeleton providing different hip assistance timings. As a limitation, future studies should consider incorporating different walking speeds, overground walking, and various surface textures to further generalize the ANN model.

## 5. Conclusions

The current study underscores the significant promise of ANN models in leveraging biomechanical data to predict metabolic costs, paving the way for advancements in robotics and rehabilitation. The precision of these models, particularly the net_GRF_ model, marks progress in creating customized assistive devices with actual metabolic data. The results indicate a bright future for ANN applications in various fields, enhancing rehabilitation technologies with diverse data and real-world feedback. Future studies could further refine these models by integrating cutting-edge technologies, like augmented and virtual reality, to improve rehabilitation and training results.

## Figures and Tables

**Table 1.** Comparative analysis of ANN parameters and performance for the GRF and moment models. This table summarizes key parameters and performance metrics, including training, validation, testing (unseen data) ratios, hidden layer sizes, input delays, and performance measures, across training, validation, testing, and overall datasets, as well as accuracy with the acceptable error margin of ± 20%, recall, precision, F1 score, and k-fold cross-validation (k = 5). It highlights the predictive accuracy and correlation coefficients, illustrating the nuanced differences in model performance and efficacy.

ANN Parameters and Performance		Net_GRF_	Net_Moment_
Settings	Training ratio (%)	70	70
	Validation ratio (%)	15	15
	Test ratio (%)	15	15
	Hidden layer size (neurons)	6	3
	Delays (count of time steps)	2	1
MSE (W/kg)^2^	Training	0.0036	0.0071
	Validation	0.0064	0.0046
	Test	0.0140	0.0121
	Total trials	0.0056	0.0075
	K-fold cross-validation test (k = 5)	0.0157 ± 0.0019	0.0123 ± 0.0013
R-value	Training	0.963	0.920
	Validation	0.927	0.956
	Test	0.882	0.874
	Total trials	0.942	0.918
	K-fold cross-validation test (k = 5)	0.842 ± 0.016	0.850 ± 0.024
*p*-value	Training	<0.001	<0.001
	Validation	<0.001	<0.001
	Test	<0.001	<0.001
	Total trials	<0.001	<0.001
	K-fold cross-validation test (k = 5)	<0.001	<0.001
Performance Metrics	Accuracy (%)	81.72	77.32
	Recall (%)	83.27	77.32
	Precision (%)	81.72	77.32
	F1 score	0.82	0.77

## Data Availability

The raw data supporting the conclusions of this article will be made available by the authors on request.
